# The Effects of Long-term Molybdenum Exposure in Drinking Water on Molybdenum Metabolism and Production Performance of Beef Cattle Consuming a High Forage Diet

**DOI:** 10.1007/s12011-022-03532-9

**Published:** 2023-01-05

**Authors:** M. P. Thorndyke, O. Guimaraes, M. Medrado, H. Y. Loh, B. V. Tangredi, A. Reyes, R. K. Barrington, K. Schmidt, N. M. Tillquist, L. Li, J. A. Ippolito, J. T. Zervoudakis, J. J. Wagner, T. E. Engle

**Affiliations:** 1 Department of Animal Science, Colorado State University, Fort Collins, CO USA; 2 Department of Animal Science, Faculdade de Ciências Agrárias E Jaboticabal, Jaboticabal, Sao Paulo, Brasil; 3 Department of Animal Science, University of WI – River Falls, River Falls, USA; 4 Department of Environmental Science, Henan University of Technology, Zhengzhou, Henan China; 5 Department of Soil and Crop Science, Colorado State University, Fort Collins, CO USA; 6 Department of Animal Science, Universidade Federal de Mato Grosso, Cuiaba, Mato Grosso Brazil

**Keywords:** Copper status, Water intake, Immunity, Mineral status, Supplemented molybdenum

## Abstract

Fifty-four multiparous beef cows with calves were used to evaluate the effects of Mo source (feed or water) on reproduction, mineral status, and performance over two cow-calf production cycles (553 days). Cows were stratified by age, body weight, liver Cu, and Mo status and were then randomly assigned to one of six treatment groups. Treatments were (1) negative control (NC; basal diet with no supplemental Mo or Cu), (2) positive control (NC + Cu; 3 mg of supplemental Cu/kg DM), (3) NC + 500 µg Mo/L from Na2MoO4·2H2O supplied in drinking water, (4) NC + 1000 µg Mo/L of Na2MoO4·2H2O supplied in drinking water, (5) NC + Mo 1000-water + 3 mg of supplemental Cu/kg DM, and (6) NC + 3.0 mg of supplemental Mo/kg diet DM from Na2MoO4·2H2O. Animals were allowed ad libitum access to both harvested grass hay (DM basis: 6.6% crude protein; 0.15% S, 6.7 mg Cu/kg, 2.4 mg Mo/kg) and water throughout the experiment. Calves were weaned at approximately 6 months of age each year. Dietary Cu concentration below 10.0 mg Cu/kg DM total diet reduced liver and plasma Cu concentrations to values indicative of a marginal Cu deficiency in beef cows. However, no production parameters measured in this experiment were affected by treatment. Results suggest that Mo supplemented in water or feed at the concentrations used in this experiment had minimal impact on Cu status and overall performance.

## Introduction

Numerous experiments have investigated the impact of water quality on beef cattle production. Nitrates, sulfates, microbial contamination, and excessive cations are water contaminants that can negatively impact beef cattle production [1, 2]. As described by Thorndyke et al., certain locations within the Rocky Mountain region in the USA have natural rock formations or human activity that can contribute to elevated concentrations of molybdenum (Mo) in surface and/or groundwater that may impact livestock performance [3]. Elevated dietary Mo concentrations have been reported to reduce copper (Cu) status in ruminants [4–6]. The negative impact of Mo on Cu status in ruminants is substantially increased when elevated concentrations of sulfur (S) are added to the diet [6–10].

Limited controlled research has been conducted investigating the influence of Mo water concentrations on cattle production and Cu status. Kincaid investigated the short-term exposure (21 days) of weaned calves (5 weeks old) to varying concentrations of Mo in water (0.0, 1,000, 10,000, or 50,000 µg of Mo/L) [11]. The author reported no difference in body weight gain across all treatments and a safe ratio of total dietary Cu to Mo of 0.5:1.0. Kistner et al. conducted a longer duration experiment (approximately 131 days) utilizing beef steers with lower doses (0–960 µg Mo/L) of Mo added to the water [12]. Diets used in this study contained approximately 10.4 mg Cu/kg DM, and Mo dose had no impact on growth, animal health, Cu status, or carcass characteristics. More recently, Thorndyke et al. conducted an experiment investigating the impact of Mo in water or feed on Cu absorption in beef cattle [13]. They reported that short-term Mo exposure in water may impact Cu absorption and retention to a lesser degree than when Mo is supplemented in the diet.

The impact of prolonged exposure to elevated Mo water concentrations on beef cattle consuming a high forage-based diet is unknown, yet these systems may be realized in areas where Mo in cattle drinking water sources are due to natural or anthropogenic inputs. Therefore, the objective of the current experiment was to conduct a life-cycle production and health assessment of lactating and gestating beef cattle, and their calves, exposed to varying doses of Mo. We hypothesized that as the duration of Mo exposure increased, animal performance and Cu status would be impaired.

## Materials and Methods

### Experimental Design

Prior to the initiation of the experiment, all animal use, handling, and sampling techniques described herein were approved by the Colorado State University Animal Care and Use Committee (IACUC approval #18-7819A). The overall project lasted 553 days with the animals housed in pastures and feedlot pens, depending on the time of year (described below).

Thirty-five days prior to the initiation of the experiment, 54 multiparous commercial (Angus & Angus x Hereford) beef cows and 54 nursing calves were purchased from a local cow-calf producer in Grand County, CO, USA. Cows were stratified based on age, BW, and initial liver Mo and Cu status and then randomly assigned to one of six treatments (*n* = 9 cow-calf pairs per treatment). The study was initiated in June. Cows calved each year in February and March, and calves were weaned in October. Treatments consisted of (1) negative control (NC; basal diet with no supplemental Mo or Cu), (2) positive control (PC: NC + Cu; 3 mg of supplemental Cu/kg diet DM from CuSO4·5H2O), (3) NC + 500 µg Mo/L from Na2MoO4·2H2O supplied in drinking water (Mo 500-water), (4) NC + 1000 µg Mo/L of Na2MoO4·2H2O supplied in drinking water (Mo 1000-water), (5) NC + Mo 1000-water + 3 mg of supplemental Cu/kg diet DM from CuSO4·5H2O (Mo 1000-water + Cu), and (6) NC + 3.0 mg of supplemental Mo/kg diet DM from Na2MoO4·2H2O (3.0 Mo-diet). Total calculated intake of Cu and Mo (water and feed) expressed on a mg/kg diet DM (Cu/Mo) basis for each treatment were as follows: NC 6.8/2.6; PC 9.8/2.6; Mo 500-water 6.8/4.3; Mo 1000-water 6.8/6.0; Mo 1000-water + Cu 9.8/6.1; and 3.0 Mo-diet 6.8/5.6

All procedures described below were repeated following the weaning of the first calf crop, except where noted. Cows remained on the same treatment for the duration of the experiment, while calves were removed from treatments at the time of weaning. All cows and calves received standard vaccinations (Bovi-Shield Gold; Zoetis, Kalamazoo, MI, USA, and Covexin 8; Merck, Omaha, NE, USA) and were dewormed (Eprinex; Boehringer Ingelheim, St. Joseph, MO, USA) yearly per recommendations of the USDA APHIS–Veterinary Services Foreign Animal Disease Preparedness and Response Plan [14] in consultation with the local veterinarian in Grand County, CO, USA. Additionally, all cows and calves were tested for bovine viral diarrhea virus by obtaining an ear notch from each animal prior to the initiation of the experiment. Bovine viral diarrhea virus was not detected in any animal used in this experiment.

### Animal Housing

Each year, animals were housed in two different locations. In the summer and early fall months (northern hemisphere), animals were housed in one of 6 pastures (≈ 1.2 ha per pasture) by treatment. Pastures were located in Grand County, CO, USA, and had no standing water and minimal naturally occurring forage cover. Each pasture contained four 1650 L water tanks. Each water tank was fenced to allow only cows access to water tanks. The water utilized in this experiment was transported from the Williams Fork River to the water tanks using a water truck. Each pasture was also equipped with a creep feeder that contained two 265 L water tanks only accessible to calves within that pen. This allowed the determination of water intake for cows and calves separately within a pasture. Each pasture also contained one round bale feeder that was placed in an empty water tank to limit calves from consuming hay from the round bale feeder. Feed troughs were placed in each creep feeder to allow calves access to hay. This allowed for feed intake determination for cows and calves independently while housed together within a pasture. In year 1, cow/calf treatment groups were rotated to a new pasture location once. In year 2, pasture rotation occurred approximately every 28 days so that all cows were exposed to each pasture location. For each rotation, all waterers, feeders, and loose salt feeders were moved to the appropriate pasture in order to maintain treatment integrity.

During the winter and early spring months, all animals were transported to the Agriculture, Research, Development, and Education Center (ARDEC) feedlot in Fort Collins, CO, USA, and housed in feedlot pens (7 m × 40 m) containing 3 cow-calf pairs from the same treatment per pen (3 replicates per treatment). Each pen was equipped with a concrete feed bunk, a 3 m × 7 m concrete bunk pad, and a 1050 L water tank. Feed was delivered daily in amounts that allowed ad libitum access to feed for cow and calves.

### Feed, Supplement, and Water Delivery

For both years, grass hay was purchased from local hay producers in Grand County, CO, USA. All hay bales were sampled and analyzed for nutrient composition (Table 1). While cattle were on pasture in Grand County, CO, USA, all hay was weighed prior to being placed in the round bale feeders or feed troughs within the creep feeders. Loose white salt was also provided to all cattle while on pasture. For a given pen, loose white salt was placed in rubber troughs and hung on the outside of the creep feeder at a height to only allow cow access. Loose white salt was also placed in a rubber tub within the creep feeder to allow calves access to white salt. Supplemental Cu (as CuSO4∙5H2O) or molybdenum (Na2MoO4·2H2O) was added to the loose salt supplement for those cows and calves receiving dietary Cu or Mo treatments (Table 2). Weekly intake of white salt was determined for cows and calves. Molybdenum and Cu concentrations of the loose salt supplement, as well as the Cu:Mo ratio for each treatment were calculated using the actual intake of water, feed, and supplement consumed by cows and calves.

**Table 1:** Nutrient composition of grass hay (dry matter basis)

Nutrient	Mean	Standard deviation
Dry matter, %	86.45	5.69
Crude Protein, %	6.27	0.72
Acid detergent fiber, %	35.01	1.78
Neutral detergent fiber	57.91	3.29
Net energy for lactation, Mcal/kg	0.53	0.07
Net Energy for gain, Mcal/kg^b^	1.19	0.13
Net energy for maintenance, Mcal/kg^c^	1.06	0.08
Total digestible nutrients, %	53.47	2.69
Digestible energy, Mcal/kg	2.39	0.12
Metabolizable energy, Mcal/kg	2.09	0.14
Calcium, %	0.42	0.08
Phosphorus, %	0.14	0.04
Potassium, %	1.81	0.14
Magnesium, %	0.15	0.03
Sodium, %	0.03	0.01
Sulfur, %	0.15	0.06
Cobalt, mg/kg	0.24	0.06
Copper, mg/kg^a^	6.84	2.91
Iron, mg/kg	99.23	14.42
Manganese, mg/kg	180.11	20.12
Molybdenum, mg/kg	2.58	0.26
Selenium, mg/kg	< 1.50	–-
Zinc, mg/kg	20.54	1.02

^a^Copper inclusion to copper-containing treatments was adjusted based on the copper concentration of each hay source. Samples (*n* = 525) were included in this analysis

^b^*NEg* = {[1.42 × (*TDN* × 0.0361)] − [0.174 × (*TDN* × 0.0361) × (*TDN* × 0.0361)] + [0.0122 × (*TDN* × 0.0361) × (*TDN* × 0.0361) × (*TDN* × 0.0361) − 1.65]} ÷ 2.205

^c^*NEm* = {[1.37 × (*TDN* × 0.0361)] − [0.0138 × (*TDN* × 0.0361) × (*TDN* × 0.0361)] + [0.0105 × (*TDN* × 0.0361) × (*TDN* × 0.0361) − 1.12]} ÷ 2.205

**Table 2:** Molybdenum (Mo) and copper (Cu) concentrations of water, white salt, and dried distillers grains (DDG) utilized to deliver experimental treatments (mean ± SD)

Item	Treatment					
	Negative control^a^	Positive control^b^	500 µg Mo/L H_2_O^c^	1000 µg Mo/L H_2_O^d^	1000 µg Mo/L H_2_O + dietary Cu^e^	Mo diet^f^
Water						
Mo, µg/L	< 10.0	< 10.0	531.4 ± 22.47	1037.0 ± 117.62	1087.3 ± 121.12	< 10.0
Cu, µg /L	< 10.0	< 10.0	< 10.0	< 10.0	< 10.0	< 10.0
White salt^g^						
Mo, mg/kg DM	< 0.01	< 0.01	< 0.01	< 0.01	< 0.01	458.21 ± 20.34
Cu, mg/kg DM	< 0.01	441.43 ± 30.31	< 0.01	< 0.01	445.71 ± 29.27	< 0.01
DDG^h^						
Mo, mg/kg DM	0.91 ± 0.07	0.89 ± 0.06	0.94 ± 0.10	0.87 ± 0.14	0.92 ± 0.13	161.11 ± 10.18
Cu, mg/kg DM	6.21 ± 0.14	158.12 ± 12.20	6.29 ± 0.18	6.18 ± 0.12	160.18 ± 10.25	6.07 ± 0.19
Cu:Mo ratio^i^	2.63:1	3.79:1	1.56:1	1.14:1	1.59:1	1.21:1

^a^Negative control: no supplemental Mo or Cu added to the diet or water

^b^Positive control: 3.0 mg Cu/kg DM from CuSO_4_·5H_2_O added to the basal diet

^c^500 µg Mo/L H_2_O: negative control diet + 500 µg Mo/L from Na_2_MoO_4_·2H_2_O supplied in the drinking water

^d^1000 µg Mo/L H_2_O: negative control diet + 1000 µg Mo/L of Na_2_MoO_4_·2H_2_O supplied in the drinking water

^e^Mo 1000-water plus 3 mg Cu/kg DM from CuSO_4_·5H_2_O added to the basal diet

^f^Mo diet: negative control diet plus 3.0 mg Mo/kg DM from Na_2_MoO_4_·2H_2_O added to the basal diet

^g^Target intake was 0.7% of DM intake

^h^Dried distillers grains; supplemented at 0.25 kg∙animal^−1^∙day^−1^ or 1.9% of diet DM and contained 0.48% sulfur

^i^Calculated based on the actual intake of water, feed, and supplement consumed by cows in this experiment

Due to the low nutrient quality of the hay being fed, a custom molasses-based protein hard lick-tub supplement (30% CP on a DM basis; 16% equivalent CP units from urea; and 14% CP units from soybean meal) was formulated to supply the appropriate amount of protein and vitamins A, D, and E for gestating and lactation beef cattle with a targeted intake of 0.34–0.57 kg∙animal^−1^∙day^−1^). Supplemental protein contained no added Cu and was provided to all cattle in tubs throughout the duration of the experiment. Protein intake was quantified weekly by weighing the protein tub. All cows and calves within a pen had ad libitum access to the same protein tub.

After transporting cattle to the ARDEC facility in Fort Collins, CO, USA, dried distillers grains (DDG) were used as the carrier for all Mo and Cu dietary treatments. Dried distillers grains were added to each pen daily (0.25 kg animal/day) at the time of hay delivery. Cattle on treatments not receiving supplemental Mo or Cu were fed the same amount of DDG without additional Mo or Cu (Table 2). The same hay fed to cattle in pastures was fed at the ARDEC facility. Water intake was monitored twice a week by measuring the disappearance of water over a given time period as described by Kistner et al. [12]. Sodium molybdate dehydrate (Na2MoO4·2H2O) was added to each water tank at the appropriate concentration, via a dilution of a 65,000 µg of Mo/L stock solution. Water Mo and Cu concentrations for each treatment over the entire experiment are shown in Table 2.

### Animal Sampling

For all cows and calves, BW and jugular blood samples were obtained approximately every 28 days. Blood samples were collected into three 7 ml vacutainer tubes ((1) heparinized trace-mineral-free vacutainer tubes, (2) vacutainer tubes containing EDTA, and (3) vacutainer tube containing no additive; Becton Dickinson Co., Franklin Lakes, NJ, USA). Once collected, blood samples were placed on ice and transported back to the laboratory (approximate time from collection until processing was 6 h). Heparinized trace-mineral-free vacutainer tubes and vacutainer tubes with no additive were centrifuged at 2000 × g for 15 min at room temperature, and plasma or serum was then transferred to acid-washed storage vials and stored at − 20 °C. One milliliter of red blood cells (RBC) from each tube was lysed in 4 mL of cold deionized H2O and stored at − 80 °C until superoxide dismutase activity analysis could be performed. Vacutainer tubes containing EDTA were submitted to the Colorado State University Diagnostic Laboratory for complete blood count (CBC) determination. Whole blood was also analyzed for pH, pCO2, and pO2 using an I-STAT blood chemistry device.

Milk samples (approximately 50 mL) were obtained from all cows at approximately 3-month post calving, for both years, by placing each cow in a squeeze chute and manually hand milking each cow. Liver biopsies (approximately 150 mg wet weight) were obtained from all cows at the beginning of the experiment, prior to transport to the ARDEC facilities, prior to transport back to pastures in Grand County, CO, USA, and prior to transport back to ARDEC in the second year of the experiment [15, 16]. Liver samples (approximately 100 g wet weight) were also obtained from the right lobe of the liver from all cows at the time of slaughter. Calf liver biopsies were obtained from all calves at approximately 3 months of age for year 1 calves and 1.5 months of age for year 2 calves.

Once weaned, all calves were removed from their respective treatments, commingled by sex, and fed a standard feedlot finishing diet for approximately 220 days. On the day of slaughter, cattle were transported to a commercial abattoir, and individual carcass data and liver samples were collected. Hot carcass weight was determined at the time of slaughter. Carcasses were allowed to chill for approximately 36 h. Standard carcass data measurements were collected by trained personnel. After weaning the second calf crop, all cows were slaughtered as described above. Kidney, muscle (longissimus dorsi), pancreas, spleen, and subcutaneous fat were obtained post slaughter and analyzed for Mo and Cu concentrations.

### Cow Reproductive Performance

To determine the effects of treatments on cow reproductive performance, every cow was inseminated once following a modified Select-Synch method [17] with the addition of a controlled internal drug-release insert (CIDR) estrus synchronization protocol as described by Ahola et al. [18]. Cows and calves were then returned to their appropriate pastures. Fourteen days after mass insemination, six Angus bulls, that had passed breeding soundness exams, were housed (1 per pasture) with the cows and calves for 60 days (1 bull per pasture). Pregnancy was determined at 40 days after mass insemination via rectal ultrasonography to classify fetuses as either artificial insemination pregnancies or natural service pregnancies.

#### Cow and Calf Performance

Cow body weights and body condition scores (BCS; 1 = emaciated, 9 = obese; [19]) were collected at approximately 28-day intervals throughout the experiment. Over both years, body weight gain and 205 days adjusted weaning weights were collected to evaluate calf performance.

### Health Status, Immune Parameters, and Blood Chemistry

Throughout the entire experiment, all animals were visually monitored daily to detect signs of morbidity by trained personnel as described by Caldera et al. [20]. Necropsies were performed on animals that died during the experiment (*n* = 3 cows and *n* = 6 calves). Briefly, 3 cows died during the experiment. One cow from the 3.0 Mo-diet died on day 1 of the experiment due to bloat, and one cow from the same treatment (3.0 Mo-diet) was euthanized on day 401 of the experiment due to bovine traumatic reticulopericarditis (hardware disease). A cow receiving the Mo 500-water treatment was euthanized on day 168 of the experiment because of loss of teeth. Six calves died during the course of the experiment. A calf on the PC treatment died of respiratory disease; a calf from the NC died, but the cause of death was not able to be determined. Three calves in the Mo 500-water treatment died: one calf was inadvertently separated from its mother during a winter storm and died of failure to thrive; one calf was stillborn, and one calf was stepped on by its dam during calving and died of a crushed thorax, and one calf receiving Mo 1000-water died of respiratory disease. In order to assess immune function, interferon-gamma concentrations, total IgG and IgM concentrations, RBC superoxide dismutase (SOD) enzyme activity, complete blood counts (CBC), pH, pO2, and pCO2 were determined on blood samples collected on day 0 and approximately every 28 days throughout the experiment on all cows and calves.

### Analytical Procedures

Feed samples were analyzed for moisture using the AOAC [21] Official Method 950.46 moisture removal process; CP using the AOAC [21] Official Method 992.15 (TruSpec CN, 2004); ash using the ash oven method described in the AOAC [21] Official Method 920.153; and acid and neutral detergent fiber [22]. Feed, water, plasma, milk, and liver samples were wet-ashed and analyzed for Mo and Cu concentrations using inductively coupled plasma mass spectrometry (EPA 200.8, rev. 5.4, [23]; PerkinElmer; NexION 2000 B). Water quality was analyzed using standard analytical techniques [24, 25].

Complete blood counts from whole blood were determined using a Siemens Advida 120 Hematology Analyzer (Siemens Healthineers, Erlangen, Germany). Serum ceruloplasmin activity was determined using a spectrophotometric procedure described by Houchin [26]. Total serum IgG and IgM concentrations were determined using single radio immunodiffusion assay kits (VMRD 240–30 and 246–30; VMRD, Inc., Pullman, WA, USA) as described by Stabel et al. [27]. Plasma samples were analyzed for INF-ɣ concentrations using an ELISA assay (Biosource KBC1231, Biosource International, Inc., Camarillo, CA, USA). The assay was designed as a qualitative assay but for this study was modified into a quantitative assay. Briefly, a positive control that was supplied with the kit was diluted with a negative control to make standards of known concentrations. A standard curve was created using linear regression to quantitate the INF-ɣ concentrations in the unknown samples. The regression coefficient of the standard curve was 0.991, and samples were reported as log10. Lysed RBC were analyzed for SOD activity using a SOD 525™ Assay Kit (Biotech® 21,010; Oxis Health Products, Inc., Portland, OR, USA). Superoxide dismutase activity was expressed as SOD activity per milligram hemoglobin. Hemoglobin concentration was determined using a Total Hemoglobin Assay Kit (Sigma 525-A; Sigma-Aldrich, St. Louis, MO, USA). Blood chemistry (pH, pO2, and pCO2) were analyzed using an i-STAT analyzer (Abbott, North America, Orlando FL, USA).

### Statistical Analysis

Cow and calf performance data, mineral status, nutrient analysis, and immune measurements were assessed using a restricted maximum likelihood-based, mixed-effects model, repeated-measures analysis (PROC MIXED; SAS Inst., Inc., Cary, NC, USA) where appropriate. Initial cow performance and mineral status models contained fixed effects of treatment, time, and treatment × time interaction. Initial calf performance models included fixed effects of treatment, year, age of dam, age of calf, sex of calf, and all relevant two- and three-way interactions. A spatial power covariance structure was used in the analysis, and the containment approximation was used to calculate denominator degrees of freedom. Pen was considered the experimental unit for all response variables measured. Reproductive response data were analyzed using logistic regression (PROC GENMOD of SAS). Initial models for reproductive response contained fixed effects of treatment, BCS, BW, and year, in addition to all relevant two- and three-way interactions. When an interaction was not significant, it was removed from the model. If the interaction of year × treatment was not significant, data were pooled across years; otherwise, data were reported for each year separately. Significance was determined at *P* ≤ 0.05, and tendencies were determined if *P* > 0.05 and ≤ 0.10.

## Results and Discussion

### Feed and Water

Table 1 describes the chemical composition of the grass hay used throughout the experiment. There were no year by grass hay interactions for any of the components analyzed. Therefore, all data are presented as overall means ± standard deviations. The basal grass hay used in this experiment was a low-quality grass hay typically fed to gestating and lactating beef cows and calves and contained 6.84 ± 2.91 and 2.58 ± 0.26 mg of Cu and Mo/kg diet DM, respectively.

Molybdenum and Cu concentrations in the water, white salt, and DDG supplements are shown in Table 2. Targeted concentrations for Mo in the Mo water treatments were 500, 1000, and 1000 mg/L for treatments Mo 500-water, Mo 1000-water, and Mo 1000-water + Cu, respectively. Analyzed Mo water concentrations were slightly greater than the targeted values for all Mo water treatments. Molybdenum and Cu concentrations were below detection limits for the basal white salt supplement and within normal concentrations for DDG [28]. Molybdenum and Cu concentrations of the white salt and DDG supplements for PC, Mo 1000-water + Cu, and the 3.0 Mo-diet treatments were formulated to supply an additional 3.0 mg of Mo and/or Cu/kg total diet DM (pasture targeted salt intake was 0.7% of DM intake; feedlot targeted DDG intake was 0.25 kg∙animal^−1^∙day^−1^ or 1.9% of diet DM). Overall, total dietary Cu concentrations were slightly below (9.8 mg Cu/kg DM) targeted NASEM [28] Cu intake concentrations of 10.0 mg Cu/kg DM for the PC and Mo 1000-water + Cu treatments, whereas Mo concentrations in the 3.0 Mo-diet treatment were slightly above (3.1 mg Mo/kg DM) the targeted supplemental intake of 3.0 mg Mo/kg diet DM. The Cu:Mo ratio was calculated based on the actual intake of water, feed, and supplements consumed by the cows throughout the experiment. The Cu:Mo ratios of the NC, PC, Mo 500-water, Mo 1000-water, Mo 1000-water + Cu, and 3.0 Mo-diet based on forage and supplement intake were 2.63:1, 3.79:1, 1.56:1, 1.14:1, 1.59:1, and 1.21:1, respectively (Table 2).

Water hardness, nitrate concentrations, and total dissolved solids were within the “safe and should pose no health problems” category for beef cattle for all water sources used (Table 3) [28]. Molybdenum and Cu water concentrations were below detection limits in both water sources at all sampling timepoints.

**Table 3:** Chemical composition of water supplied to cattle while housed in Grand County, CO, USA (Williams Fork River) and at the Agricultural, Research, Development, and Education Center (ARDEC-well water)

Item	Williams Fork River water Result (mean ± standard deviation)	ARDEC-well water Result (mean ± standard deviation)
*n* =	115	126
pH, s.u	7.24 (± 0.17)	7.42 (± 0.16)
Chloride, mg/L	5.2 (± 0.10)	28.6 (± 3.24)
Total Hardness, mg/L	26.4 (± 1.21)	75.4 (± 58.24)
Nitrate-Nitrogen, mg/L	< 1.0 (± N/A)	< 1.0 (± N/A)
Calcium, mg/L	7.93 (± 0.21)	174.2 (± 12.54)
Magnesium, mg/L	1.53 (± 0.06)	54.23 (± 0.071)
Phosphorous, mg/L	< 0.1 (± N/A)	< 0.1 (± N/A)
Potassium, mg/L	< 5.0 (± N/A)	< 5.0 (± N/A)
Sodium, mg/L	< 5.0 (± N/A)	56.3 (± 9.29)
Sulfate, mg/L	2.72 (± 0.08)	432.7 (± 29.70)
Aluminum, mg/L	0.21 (± 0.01)	0.06 (± 0.004)
Cobalt, mg/L	< 0.01 (± N/A)	< 0.01 (± N/A)
Copper, µg/L	< 10.0 (± N/A)	< 10.0 (± N/A)
Iron, mg/L	0.27 (± 0.01)	0.12 (± 0.02)
Manganese (mg/L)	< 0.01 (± N/A)	0.08 (± 0.05)
Molybdenum (µg/L)	< 10.0 (± N/A)	< 10.0 (± N/A)
Selenium (µg/L)	< 30.0 (± N/A)	< 30.0 (± N/A)
Total dissolved solids (mg/L)	42.5 (± 3.97)	985.7 (± 85.24)

Due to the low protein content of the hay, a free choice molasses-based protein hard lick-tub supplement was formulated to meet the NASEM [28] protein requirements for mature lactating and gestating cows with a targeted intake of 0.34–0.57 kg∙animal^−1^∙day^−1^. No supplemental Cu was added to the protein supplement. The Mo and Cu content of the protein supplement was 0.67 and 2.34 mg/kg DM, respectively. Overall protein intake was 0.41, 0.39, 0.44, 0.43, 0.38, and 0.42 (SEM = 0.06) kg∙animal^−1^∙day^−1^ for NC, PC, Mo 500-water, Mo 1000-water, 1000-water + Cu, and 3.0 Mo-diet treatments, respectively (data not shown).

### Cow Performance and Mo and Cu Status

Morbidity and mortality rates were low throughout the experiment, and no signs of Mo toxicity were observed. The influence of chronic molybdenum exposure in drinking water or feed on cow performance and water intake over the duration of the experiment is shown in Table 4. There were no treatment × time interactions for any cow performance response variables. Therefore, data were pooled across year. Body weights, DM intake, water intake, and overall pregnancy rates were similar across treatments and within normal ranges for moderately framed beef cows [28].

**Table 4:** The influence of chronic molybdenum exposure in drinking water or feed on cow performance

Item	Treatment						SEM	*P* <		
	Negative control^a^	Positive control^b^	500 µg Mo/L H_2_O^c^	1000 µg Mo/L H_2_O^d^	1000 µg Mo/L H_2_O + dietary Cu^e^	Mo diet^f^		TRT	Time	Trt × time
Initial body weight, kg	522.5	571.3	529.0	548.6	540.9	579.5	12.3	0.72	–-	–-
Final body weight, kg	553.1	557.4	561.2	554.9	562.3	561.7	8.9	0.64	0.001	0.59
Body condition score^g^	4.9	5.1	5.3	5.0	5.1	5.2	0.05	0.81	0.0001	0.83
Dry matter intake, kg/d	13.6	14.8	14.2	14.4	13.8	14.0	1.3	0.73	0.05	0.68
Water intake, L∙animal^−1^∙d^−1^	44.2	45.3	46.9	46.9	45.1	46.9	7.2	0.54	0.001	0.81
Pregnancy rate to artificial insemination, %										
Year 1	56	44	56	44	56	57	–-	0.89	–-	–-
Year 2	44	56	56	44	56	71	–-	0.56	–-	–-
Overall pregnancy rate after a 60-d breeding season, %										
Year 1	100	100	89	100	100	88	–-	0.82	–-	–-
*n* =	9	9	8	9	9	7	–-	–-	–-	–-
Year 2	100	89	100	89	89	100	–-	0.79	–-	–-
*n* =	9	9	8	9	9	7	–-	–-	–-	–-

^a^Negative control: no supplemental Mo or Cu added to the diet or water

^b^Positive control: 3.0 mg Cu/kg DM from CuSO_4_·5H_2_O added to the basal diet

^c^500 µg Mo/L H_2_O: negative control diet + 500 µg Mo/L from Na_2_MoO_4_·2H_2_O supplied in the drinking water

^d^1000 µg Mo/L H_2_O: negative control diet + 1000 µg Mo/L of Na_2_MoO_4_·2H_2_O supplied in the drinking water

^e^Mo 1000-water plus 3 mg Cu/kg DM from CuSO_4_·5H_2_O added to the basal diet

^f^Mo diet: negative control diet plus 3.0 mg Mo/kg DM from Na_2_MoO_4_·2H_2_O added to the basal diet

^g^BCS: 1 = emaciated; 9 = obese[19]

The influence of chronic Mo exposure in drinking water or feed on cow liver, plasma, and milk Mo and Cu concentrations and plasma ceruloplasmin activity over the duration of the experiment is shown in Table 5. Initial and final liver Mo concentrations and initial liver Cu concentrations were similar across treatments. Final liver Cu concentrations were greater (*P* < 0.05) in cows receiving supplemental Cu (PC and 1000 Mo-water + Cu treatments) when compared to cows not receiving supplemental Cu. At the beginning of the experiment, initial liver Cu concentrations were slightly above concentrations indicative of a marginal Cu deficiency (deficiency defined as less than 20–30 mg Cu/kg) [29]. Final liver Cu concentrations were adequate for cattle receiving supplemental Cu and deficient in all other treatments.

**Table 5:** The influence of chronic molybdenum exposure in drinking water or feed on cow liver, plasma, and milk molybdenum (Mo) and copper (Cu) concentrations and serum ceruloplasmin activity

Item								*P* <		
	Negative control^a^	Positive control^b^	500 µg Mo/L H_2_O^c^	1000 µg Mo/L H_2_O^d^	1000 µg Mo/L H_2_O + dietary Cu^e^	Mo diet^f^	SEM	TRT	Time	Trt × time
Liver										
Mo, mg/kg DM										
Initial	3.62	4.44	4.07	4.14	2.86	2.93	0.63	0.94	–-	–-
Final	3.94	4.19	3.79	3.98	3.61	4.09	0.49	0.68	0.05	0.37
Cu, mg/kg DM										
Initial	31.2	30.9	31.7	32.1	32.7	32.2	1.08	0.83	–-	–-
Final^g^	24.3^x^	70.2^y^	26.3^x^	27.1^x^	61.9^y^	26.3^x^	1.23	0.01	0.001	0.01
Plasma										
Mo, mg/L										
Initial	0.17	0.16	0.20	0.21	0.22	0.18	0.04	0.92	–-	–-
Final	0.19	0.22	0.18	0.24	0.19	0.23	0.05	0.87	0.95	0.98
Cu, mg/L										
Initial	0.96	1.02	0.93	1.20	0.95	1.11	0.15	0.91	–-	–-
Final^g^	0.59^x^	1.17^y^	0.62^x^	0.58^x^	0.81^y^	0.53 ^x^	0.21	0.02	0.01	0.04
Serum ceruloplasmin, IU/L										
Initial	32.12	35.68	34.94	34.17	36.90	35.43	0.89	0.78	–-	–-
Final^g^	10.32^x^	40.21^y^	12.71^x^	11.39^x^	41.47^y^	10.97^x^	1.02	0.01	0.001	0.65
Milk										
Mo, mg/L										
Initial	0.15	0.16	0.13	0.16	0.17	0.11	0.01	0.92	–-	–-
Final	0.18	0.17	0.22	0.21	0.18	0.20	0.01	0.18	0.05	0.39
Cu, mg/L										
Initial	0.081	0.084	0.092	0.063	0.059	0.064	0.001	0.76	–-	–-
Final	0.086	0.091	0.083	0.079	0.077	0.080	0.001	0.57	0.19	0.61

^a^Negative control: no supplemental Mo or Cu added to the diet or water

^b^Positive control: 3.0 mg Cu/kg DM from CuSO_4_·5H_2_O added to the basal diet

^c^500 µg Mo/L H_2_O: negative control diet + 500 µg Mo/L from Na_2_MoO_4_·2H_2_O supplied in the drinking water

^d^1000 µg Mo/L H_2_O: negative control diet + 1000 µg Mo/L of Na_2_MoO_4_·2H_2_O supplied in the drinking water

^e^Mo 1000-water plus 3 mg Cu/kg DM from CuSO_4_·5H_2_O added to the basal diet

^f^Mo diet: negative control diet plus 3.0 mg Mo/kg DM from Na_2_MoO_4_·2H_2_O added to the basal diet

^g^Means within a row lacking common superscripts differ *P* < 0.05

Similarly, initial and final plasma Mo concentrations and initial plasma Cu concentrations were similar across treatments. Plasma Cu concentrations were greater (*P* < 0.05) in cows receiving supplemental Cu. Initial plasma Cu concentrations were adequate (0.6 mg Cu/L) [30] for all cattle, whereas final plasma Cu concentrations were considered deficient for cows receiving no supplemental Cu (deficiency defined as plasma Cu concentrations < 0.6 mg Cu/L) [30]. Serum ceruloplasmin activity followed a similar pattern as plasma Cu concentrations. Initial ceruloplasmin activity was similar across treatments, whereas final ceruloplasmin activity was greater (*P* < 0.01) in cows receiving supplemental Cu when compared to all other treatments.

Initial and final Mo liver concentrations were within ranges considered to be normal (0.6–6.0 mg Mo/kg DM; CSU Veterinary Diagnostic Laboratory). Initial and final plasma Mo concentrations were considered to be elevated in all treatments (0.08–10.0 mg Mo/L) [30]. Furthermore, the range of values proposed by Puls [30] indicative of “elevated” plasma Mo concentrations (0.08–10.0 mg Mo/L) is broad.

There were no treatment × time interactions for milk Mo or Cu concentrations (Table 5). Initial and final Mo and Cu concentrations in milk obtained from all cows within the experiment were similar across treatments. For all treatments, milk Mo concentrations were slightly elevated above normal (normal milk Mo concentrations = 0.018–0.120 mg Mo/L; elevated 0.200–0.400 mg Mo/L) [30], and milk Cu concentrations were within normal ranges (adequate milk Cu concentrations = 0.05–0.60 mg/L) [30].

Shorter-term experiments have been conducted examining the impact of Mo in water and feed on cattle performance. Earlier research by Kincaid indicated that short-term exposure (21 days) of 5-week-old weaned calves to different doses of Mo in water (0.0; 1000; 10,000; or 50,000 µg of Mo/L) had no impact on animal performance [11]. Based on these data, the author suggested a safe ratio of total dietary Cu to Mo to be 0.5:1.0. Kistner et al. conducted a longer duration experiment (approximately 131 days) in feedlot steers with lower doses of Mo in drinking water, ranging from 0 to 960 µg Mo/L [12]. The high concentrate feedlot diet contained 10.4 mg Cu/kg DM. The authors reported no impact on growth, animal health, Cu status, or carcass characteristics at any of the Mo doses examined in this experiment. More recently, Thorndyke et al. [13] conducted an experiment investigating the impact of Mo in water or feed on Cu absorption in beef cattle. They reported that short-term Mo exposure in water may impact Cu absorption and retention to a lesser degree than when Mo is supplemented in the diet.

Although there is limited research investigating the impact of long-term Mo supplementation on cow-calf production parameters, cow grazing studies have been conducted in areas of reclaimed mines with elevated forage Mo concentrations. Gardner et al. [31] investigated the impact of reclaimed mining area forages containing between 21 and 44 mg Mo/kg DM on cow and calf performance. Cows were allowed to graze for 12 weeks each year for three consecutive years. The authors reported no signs of Mo toxicity, Cu deficiency, or impacts on health in Cu-supplemented or non-Cu-supplemented cows. The reason for the lack of observed molybdenosis in these cattle [31] may be due to the short duration of Mo exposure. In the current experiment, animals receiving 3.0 Mo-diet showed no adverse effects from consuming a basal forage diet containing 2.58 mg/kg Mo with 3.0 mg Mo/kg DM added to the diet with a Cu:Mo ratio of 1.21:1 for greater than 500 days. Animals consuming Mo in the drinking water treatments (Mo 500-water; Mo 1000-water) with no added Cu also exhibited no adverse effects based on a Cu:Mo ratio of (1.56 and 1.14: 1, respectively) over the duration of the experiment. This suggests that a lower Cu:Mo ratio may be safe for cattle.

### Immune and Blood Chemistry Parameters in Cows

Long-term Mo exposure in drinking water or diet did not affect CBC, blood pH, pO_2_, PCO_2_, RBC SOD activity, or concentrations of interferon gamma, total IgG, and IgM (data not shown). The impact of Cu deficiency on beef cattle immunity has been variable. In vitro neutrophil killing ability of C. *albicans* [32, 33] and *S. aureus* [34, 35] were reduced in Cu deficient or marginally Cu-deficient cattle, respectively. Supplementing Cu improved the neutrophil killing ability of *C. albicans* of previously Cu-deficient calves, indicating that Cu may play a role in neutrophil function [32]. However, Jones and Suttle [32] and Boyne and Arthur [33] reported that Cu deficiency had no impact on the phagocytosis of yeast by neutrophils. Lymphocyte function, cytokine production, and antibody production in Cu-deficient cattle have been reported to be reduced [34, 36–38] or not impacted [27, 36, 37, 39–42] when compared to Cu-adequate cattle in each respective experiment. In the current experiment, it appears that Cu status was not low enough in non-Cu-supplemented cattle to impact any of the immune parameters measured.

### Calf Performance and Mo and Cu Status

Table 6 shows the effects of long-term Mo exposure in drinking water or diet on the performance of both calf crops throughout the experiment. Year 1 and 2 initial and final BW, DMI, average daily gain (ADG), and water intake were similar across treatments and are representative of typical calf growth rates and water intake [28].

**Table 6:** The effects of long-term molybdenum exposure in drinking water or diet on the performance of two calf crops from dams fed a high forage diet^1^

Item	Treatment^1^						SEM	*P* <
	Negative control^a^	Positive control^b^	500 µg Mo/L H_2_O^c^	1000 µg Mo/L H_2_O^d^	1000 µg Mo/L H_2_O + dietary Cu^e^	Mo diet^f^		Trt
Initial body weight, kg								
Year 1	138.9	153.3	146.0	136.6	153.7	143.5	2.9	–-
Year 2^ g^	33.1	34.2	32.7	31.8	32.9	33.1	0.92	0.67
Final body weight, kg^h^								
Year 1	170.5	166.8	165.6	160.4	172.7	161.4	3.0	0.74
Year 2	180.2	172.3	171.9	174.6	173.8	170.9	3.4	0.67
Dry matter intake, kg∙animal^−1^∙d^−1^								
Year 1	4.4	3.5	3.9	3.9	3.8	4.1	0.29	0.57
Year 2	4.6	4.2	4.3	4.4	4.6	4.2	0.31	0.49
Average daily gain, kg∙animal^−1^∙d^−1^								
Year 1	0.96	0.79	1.06	1.12	0.87	0.82	0.18	0.62
Year 2	1.02	0.97	0.96	1.1	1.0	0.91	0.14	0.47
Water intake, L∙animal^−1^∙d^−1,I^								
Year 1	5.2	5.7	4.6	6.0	5.5	5.9	0.39	0.58
Year 2	6.1	5.9	6.2	5.7	5.9	5.4	0.29	0.49

^1^Data presented are from birth or experiment initiation up to weaning

^a^Negative control: no supplemental Mo or Cu added to the diet or water

^b^Positive control: 3.0 mg Cu/kg DM from CuSO_4_·5H_2_O added to the basal diet

^c^500 µg Mo/L H_2_O: negative control diet + 500 µg Mo/L from Na_2_MoO_4_·2H_2_O supplied in the drinking water

^d^1000 µg Mo/L H_2_O: negative control diet + 1000 µg Mo/L of Na_2_MoO_4_·2H_2_O supplied in the drinking water

^e^Mo 1000-water plus 3 mg Cu/kg DM from CuSO_4_·5H_2_O added to the basal diet

^f^Mo diet: negative control diet plus 3.0 mg Mo/kg DM from Na_2_MoO_4_·2H_2_O added to the basal diet

^g^Birth weight for year 2 calves. Year 1 calves were approximately 5 months of age at the initiation of the experiment

^h^Adjusted 205-day weaning weight

^i^Measured during the pasture phase only

The influence of chronic Mo exposure in drinking water or feed on calf liver and plasma Mo and Cu concentrations and serum ceruloplasmin activity of both calf crops is shown in Table 7. Initial and final liver and plasma Mo and Cu concentrations and ceruloplasmin activity were similar across treatments.

**Table 7:** The influence of chronic molybdenum exposure in drinking water or feed on calf liver and plasma molybdenum and copper concentrations and serum ceruloplasmin activity of two calf crops from dams fed a high forage diet

Item								P <		
	Negative control^a^	Positive control^b^	500 µg Mo/L H_2_O^c^	1000 µg Mo/L H_2_O^d^	1000 µg Mo/L H_2_O + dietary Cu^e^	Mo diet^f^	SEM	TRT^g^	Time	Trt × time
Liver Mo, mg/kg DM										
Year 1-Initial	3.05	3.40	3.25	5.19	5.64	4.24	0.98	0.39	–-	–-
Year 1-Final	4.91	4.76	5.01	5.24	5.19	4.69	0.81	0.76	0.61	0.62
Year 2-Initial	5.31	5.28	4.97	5.47	5.21	5.63	0.94	0.58	–-	–-
Year 2-Final	5.01	5.21	5.07	5.41	4.98	5.14	0.86	0.68	0.39	0.84
Liver Cu, mg/kg DM										
Year 1-Initial	52.14	49.73	53.61	52.76	54.47	50.29	1.78	0.74	–-	–-
Year 1-Final	44.25	54.17	46.98	47.96	45.39	49.18	2.36	0.43	0.01	0.67
Year 2-Initial	103.03	120.41	106.21	111.32	117.12	108.65	4.12	0.49	–-	–-
Year 2-Final	52.97	63.02	54.01	53.64	60.98	57.36	2.31	0.64	0.41	0.54
Plasma Mo, mg/kg DM										
Year 1-Initial	0.17	0.19	0.18	0.19	0.16	0.13	0.07	0.90	–-	–-
Year 1-Final	0.19	0.17	0.20	0.16	0.17	0.21	0.08	0.67	0.81	0.93
Year 2-Initial	0.19	0.16	0.21	0.18	0.19	0.22	0.07	0.74	–-	–-
Year 2-Final	0.21	0.22	0.18	0.20	0.23	0.24	0.08	0.34	0.59	0.61
Plasma Cu, mg/kg DM										
Year 1-Initial	0.70	0.76	0.67	0.65	0.71	0.57	0.19	0.85	–-	–-
Year 1-Final	0.79	0.82	0.87	0.79	0.82	0.72	0.23	0.91	0.05	0.69
Year 2-Initial	0.72	0.81	0.69	0.75	0.79	0.70	0.19	0.64	–-	–-
Year 2-Final	0.70	0.83	0.65	0.71	0.77	0.65	0.14	0.41	0.19	0.39
Serum ceruloplasmin, IU/L										
Year 1-Initial	29.93	28.70	31.94	30.47	29.78	31.87	1.74	0.89	–-	–-
Year 1-Final	31.24	32.98	34.39	35.74	32.68	31.93	1.31	0.87	0.01	0.76
Year 2-Initial	30.21	31.63	29.98	30.87	31.07	30.22	1.21	0.64	–-	–-
Year 2-Final	31.03	32.61	29.07	29.96	31.24	30.97	1.07	0.74	0.65	0.81

^a^Negative control: no supplemental Mo or Cu added to the diet or water

^b^Positive control: 3.0 mg Cu/kg DM from CuSO_4_·5H_2_O added to the basal diet

^c^500 µg Mo/L H_2_O: negative control diet + 500 µg Mo/L from Na_2_MoO_4_·2H_2_O supplied in the drinking water

^d^1000 µg Mo/L H_2_O: negative control diet + 1000 µg Mo/L of Na_2_MoO_4_·2H_2_O supplied in the drinking water

^e^Mo 1000-water plus 3 mg Cu/kg DM from CuSO_4_·5H_2_O added to the basal diet

^f^Mo diet: negative control diet plus 3.0 mg Mo/kg DM from Na_2_MoO_4_·2H_2_O added to the basal diet

^g^There are no significant differences between means within a row at *P* < 0.05

### Immune and Blood Chemistry Parameters in Calves

The effects of long-term Mo exposure in drinking water or diet on CBC, blood pH, pO_2_, pCO_2_, and immune parameters were determined for all calves throughout the experiment (data not shown). No treatment × year or treatment × time interactions were detected for any blood chemistries or immune measurements in calves. All blood chemistry parameters (CBC, blood pH, pO2, and pCO_2_) were similar across treatments and were within normal values for beef calves. Initial and final IgG, IgM, and INFɤ were similar across treatments for years 1 and 2. Initial and final RBC SOD activity was similar across treatments in year 1. There was a tendency (*P* < 0.07) for year 2 calves receiving PC and Mo 1000-water + Cu treatments (Cu-supplemented treatments) to have lower SOD activity when compared to all other treatments (data not shown).

### Cow and Calf Slaughter Data

At the end of the experiment, cows were transported to a commercial slaughter facility. Kidney, muscle (longissimus dorsi), pancreas, spleen, and subcutaneous adipose tissue were obtained post slaughter and analyzed for Mo and Cu concentrations for all cows (data not shown). Kidney Mo concentrations were greater (*P* < 0.05) in animals receiving supplemental Mo when compared to non-Mo-supplemented cattle (*NC* = 0.62; *PC* = 0.58; 500 Mo-water = 0.73; 1000 Mo-water = 0.71; 1000 Mo-water + Cu = 0.81; Mo-diet = 1.08 mg Mo/kg DM). Furthermore, kidney Mo concentrations were greater (*P* < 0.05) in animals receiving Mo in their diet than when compared to animals receiving Mo in drinking water. Kidney Cu concentrations were greater in cows receiving supplemental Cu when compared to non-Cu-supplemented cows (*NC* = 4.08; *PC* = 5.12; 500 Mo-water = 4.32; 1000 Mo-water = 4.21; 1000 Mo-water + Cu = 5.01; Mo-diet = 4.08 mg Cu/kg DM). Treatment had no impact on the Mo or Cu concentrations in all other tissues collected.

All offspring from both years were removed from their respective treatments at weaning and fed a standard commercial finishing diet until they reached an appropriate slaughter weight. Animals were then transported to a commercial abattoir and slaughtered using standard US beef industry practices and USDA/Food safety inspection service criteria, and individual carcass data were collected. Hot carcass weight, dressing percentage, yield grade, and marbling score were similar across treatment for all calves across both years (data not shown).

The lack of a Mo impact on the response variables measured in the current experiment is similar to data reported by Gardner et al. [31] and Raisbeck et al. [43] when variable doses of Mo were consumed in feed. However, to our knowledge, the current experiment is the first experiment to examine the impact of long-term Mo supplementation in drinking water or feed on life-cycle production parameters, health, and Mo and Cu status of gestating and lactating beef cows and their calves consuming a low-quality forage diet. Under the conditions of the current experiment, no signs of molybdenosis were observed. Cattle that received diets containing less than the NASEM [28] (10 mg Cu/kg DM) Cu requirement for beef cattle became Cu deficient over the course of the experiment, as determined by liver and plasma Cu concentrations, regardless of Mo treatment. However, no Cu deficiency signs (e.g., reduced growth rate, reproductive performance, or immune function) were observed throughout the course of the experiment. Furthermore, offspring Cu status was not different across treatments. This indicates that adequate maternal transfer of Cu occurs when dams are marginally deficient in Cu in the current experiment.

Although the current experiment was designed to examine the impact of Mo in feed or water on Cu status in beef cattle, it is important to note that dietary S (feed and water) can influence the impact of Mo on Cu metabolism in ruminants. Dick was the first to determine that elevated dietary sulfur can greatly influence the antagonistic impact of Mo on Cu metabolism in ruminants [7]. Subsequent research determined that S and Mo form thiomolybdates under the reducing conditions of the rumen and can bind to Cu in the gastrointestinal tract and either prevent Cu absorption or reduce the availability of Cu once absorbed into the bloodstream [8, 9, 44–47]. In the current experiment, the S content of the diet was slightly above the NASEM [28] dietary requirement of 0.15% for ruminants and well below dietary (0.30–0.50% S) and water (333 mg S/L of drinking water) concentrations know to impact Cu metabolism in ruminants.

## Data Availability

All data would be available if requested for review.
